# A New Method of Employing Charcoal in the Treatment of Acute Indigestion in Horses

**Published:** 1899-01

**Authors:** George J. Goubeaud

**Affiliations:** Borough of Brooklyn


### A NEW METHOD OF EMPLOYING CHARCOAL IN THE TREATMENT OF ACUTE INDIGESTION IN HORSES.[1]

By George J. Goubeaud, D.V.S.,
BOROUGH OF BROOKLYN.

Upon request of one of your honored members I have the pleasure of presenting for your consideration a subject entitled "A New Method of Administering Charcoal for the Relief of Gaseous Distention in Acute Gastric and Gastro-intestinal Indigestion." During the past two years it has fallen to my lot to treat by this method a large number of horses suffering from this form of colic, and have secured much success, results being obtained that could not possibly be had under the old method of administering charcoal; indeed, I might state that it was the principal agent I relied upon.

We all know how serious is the condition of the animal that presents itself to us suffering from this affection: Abdomen distended to an enormous degree (although, at times, when the stomach alone is involved the distention will not be so noticeable); eructating gas as rapidly as the abnormally distended and half-

[1] Read before the December meeting of the Veterinary Medical Association of New York County.

paralyzed stomach will permit, the sound of the gas forcing itself up the œsophagus being heard several feet distant; mouth dribbling saliva, which the animal attempts to swallow, but is successful only after several ineffectual efforts; little or no gas escaping per rectum, although it strains in its efforts to force it out; peristalsis diminished, and in some cases absolutely no movement of the intestines can be heard; respiration quick and short; pulse usually soft and compressible, and numbering sixty to eighty per minute; mucous membranes cyanosed, due to pressure of the distended intestines upon the lungs, thereby retarding proper oxidation; body swaying from side to side; trembling or shivering of the muscles of the flank and elbow; skin covered with a cold sweat; unable to stand, and still unable to lie in the recumbent position, and an anxious countenance.

The foregoing is a picture of a case of indigestion that demands active and energetic treatment; medication that will be prompt, rapid, effectual, positive, and reliable in results. We know in cases of this kind that active and energetic measures must be employed to stop fermentation, thus preventing gaseous accumulation, and the next step is to rid the intestinal organs of the gases already formed by expulsion by means of powerful cathartics, enterotomy, or absorption. We know that the animal's condition demands such treatment, and with a failure to employ it how liable the stomach is, or some of the intestines, to rupture, and that an almost necessarily fatal termination will be the result. Our first thought after enterotomy has been performed (that is, if we find such a procedure necessary) is either to stop or prevent fermentation and to absorb the gases which have already formed. Holding views at variance with those generally accepted concerning the prevention of fermentation, and since the only object of this paper is to deal with the absorption of gases, I will proceed with the subject proper.

While attending college I listened more or less attentively to my learned and honored professors eulogizing the beneficial results that were to be obtained by the use of charcoal when given internally for the relief of gaseous distention due to fermentation in acute gastric and gastro-intestinal indigestion. We were told that charcoal absorbed several times its weight of gas, and the same statement is made by some of our standard authors on materia medica and chemistry; again, others say that charcoal absorbs fifteen times its weight of gas. Now, while all they have said may be true, still I do not think they treated very many bad cases of acute indigestion with good results; if they did, they treated a different class of horses from those which I have attended, or they used charcoal that had effects and results positively dissimilar to that which I employed. If they had good results and employed the treatment such as they told us, then they treated horses whose intestinal organs could not be ruptured even by a modern ram.

I employed charcoal, first vegetable, then animal, and whatever results were to be obtained by the use of the former, still I did not then, nor do I now, unless under certain conditions, believe that animal charcoal has the same absorbing powers that vegetable charcoal possesses. I gave it dry in powdered state, in capsule form, and I gave as much as three pounds to one animal, with no effect. Now, gentlemen, I had positively and absolutely no results. The only result I did get I would have obtained had I not used charcoal at all. When I did not give it I had cases recover, and they seemed to be extremely bad cases—animals that I thought would die. They presented all the symptoms of an aggravated attack—an attack which apparently would lead to a fatal termination, and still they would recover. Then, again, other cases which would not appear so bad would terminate fatally. Now, identically the same can be said of those cases which I treated with charcoal. Cases died which would have recovered had the charcoal possessed the powers with which it was credited. No fatal symptoms or fatal complications had presented themselves until two and three hours had elapsed after charcoal had been administered. If charcoal had performed its proper action, why did my cases, not those that had lesions other than those of rupture, present lesions of rupture upon post-mortem examination, if charcoal had performed the action which was ascribed to it? If charcoal absorbed several times its weight of gas, and my patient died after having received two pounds of it, then that which I gave must have been hoodooed. I do not know just now how much volume is in an ounce of gas, but I do know that it is considerable. Now if charcoal such as we prescribe will absorb several times its weight of gas, then sixteen ounces of charcoal will absorb, to make it small, three times as much, or forty-eight ounces of gas; or, still further, if three pounds of charcoal will absorb fifteen times its weight of gas, and still both horses die, due to shock from ruptured stomachs, then something must be wrong somewhere.

Two cases in particular proved to me the inefficiency of charcoal when employed as an absorbent in the manner in which it is usually administered, and I hope to be pardoned for entering into them in detail.

Case I.—An aged trotting horse; he had become unfastened and eaten one-half bushel of green corn. From the time of sickness to the period of beginning treatment was about forty minutes. I tapped him immediately, much against the owner's wishes. As other treatment I gave him charcoal, the amount which he received being two pounds. Some idea of the severity of the attack can be had when I state that it required about two hours to give him this quantity. The last time I tapped him as much gas escaped as at the first operation. The owner became dissatisfied, and so was I, at the progress his horse was making. He summarily dismissed me, and another veterinarian was called, who immediately gave him linseed oil, one quart; chloroform, ℥iij; aromatic spirits of ammonia, ℥iij; result, death one hour later. Post-mortem examination: Pulmonary medication too heroic; the major portion of the drench was in the bronchial tubes; stomach presented an incomplete rupture; the gastric peritoneum was the only structure which held the organ intact, and had the animal lived long enough I have every reason to believe that the rupture would have been complete.

Case II.—Was owned by a physician, and I think it was for that reason I gave the animal charcoal. The physician was a believer in the efficacy of it; I was not, although I gave it. On account of working hard, the groom gave the horse a double quantity of oats. He developed an attack of acute gastric indigestion half an hour afterward. Death occurred five hours after developing the attack. With other treatment which I considered appropriate at the time, I gave him one pound and a half of vegetable charcoal. I must confess I saw positively no effect from its use. The post-mortem examination revealed a complete rupture of the stomach over its greater curvature, about eight inches long, and running from right to left. That part of the organ which remained intact was empty. This result convinced the owner of the uselessness of charcoal when administered in the manner in which it was.

I might add that since then I had a case which, to all appearances, was as bad as the last mentioned, owned by the same gentleman, caused in the same way, but treated differently, which did not terminate fatally. I think that I can safely state that the owner's opinion of charcoal has changed considerably.

Still I have heard prominent and successful practitioners say that they had cases in which the gas generated as rapidly as it was absorbed by the charcoal.

Practitioners who had employed hundreds of pounds in practice, who are shrewd observers, careful, conservative, and conscientious, would have me believe that a horse will generate three times as much gas as charcoal will absorb. Others, that during fermentation a horse will evolve fifteen times as much gas as charcoal by its affinity will take to itself. If these things be true the deductions would be as follows: A horse would be an animated gas reservoir; a walking, rolling, kicking, and struggling gas generator, and an honest and faithful gas tank. I could not understand these things; I was either badly twisted or they were wrong. The authorities either know that charcoal does not, would not, and could not absorb gases under certain conditions, and failed to state the fact—for I have not found it stated in all the works which I have read on the subject—or they simply wished to have us find the reason why.

Charcoal, by its selective affinity and its powers to absorb gases, is described as an absorbent. It will, by that power which it possesses and which is inherent in itself, perform this action; it will absorb gas; it is one of the best absorbents of gas that is known. But there are conditions or states in which charcoal can be and is absolutely useless as an absorbent, and I think I can prove to your satisfaction that charcoal as we give it is positively useless, and can have no absorbing power whatever. It simply and positively has no absorbing power.

Charcoal is not very obliging; it does not wait for us to employ it and then perform its chemical action; it is not automatic; we must do more than press the button. We must heat it, we must drive off all those gases which it has already absorbed before any benefit can be derived from its use. The arguments sustaining my contention are these: that charcoal, after the heat leaves it, is stored away until needed. It is then powdered, placed in bottles, or tin or paper boxes, it makes no difference which, placed in stock until called for. From the time of manufacture to the time of use it may be from one month to one year. We place it in bottles or in our medicine drawers, or perhaps we leave it in its original package, lay it aside in a secluded corner, usually on the floor of our medicine-case, so that it can be easily reached and still out of the way. Whenever necessary we take as much as we wish, rush off, usually leaving the box or whatever receptacle it is kept in uncovered until we return. In due time it is placed away for future use.

Now, during the time of its manufacture to the time of its employment there is no reason why it will not absorb whatever gases there are in the surrounding atmosphere it comes in contact with, and if it does not absorb gases that are in an unhealthy atmosphere, it will absorb gases that are in a normal atmosphere, and irrespective of the quality of the gases, whether they be harmless or injurious. The charcoal, from the period of its manufacture to the time of its employment, has been so surcharged with gaseous matters, supersaturated, as it were, with the surrounding atmospheric constituents, extra laden with the gaseous elements, that when it is administered for the relief of gaseous distention it cannot, it will not, and it does not, absorb gases, for the reasons which I have stated, but it will absorb gas if it is properly prepared before employment. It will positively perform its proper chemical action, it will most assuredly come to our relief, and I can honestly state that I have had the most surprising and gratifying results from its use, and I can still add that if our patient has not developed some fatal lesions due to distention our case will terminate favorably.

I could recite case after case in which the results were most astonishing. The action of it was rapid, effectual, and positive; but we must first do one thing, and that is to drive off all those gases that are in the charcoal. We can do that by heat. Heat the charcoal, and when it is sufficiently cool to handle place in capsules as quickly as possible and give immediately. The quantity which I usually give is four ounces. A very neat way to give it, and also to have it ready for immediate use, is to heat the charcoal until red hot, fill capsules, cover them, place in wide-mouthed bottles, cover tops of capsules with cotton so as to prevent caps from coming off while being carried, cork bottles securely, seal with wax, and store away in a dry place for future use. The bottle must be hermetically sealed.

To those wishing to prove for themselves the correctness of my views by a personal comparison and practical test, I will simply state that if they will collect any gas in a glass retort, place a piece of charcoal in the retort and note the result, I doubt little if any gas will be absorbed by the charcoal, the amount depending upon the freshness of the charcoal. Now heat the same piece of charcoal, place in the retort with gas, and note the result. I hardly think any gas will remain in the retort. I have experimented further, but this experiment will be sufficient to establish my claim. It is not only necessary for us to heat the charcoal until it is too hot to handle; we must heat it till red hot, allow time to cool sufficiently, place in capsules, cork them, and in a very short time the internal heat will pass off, so that it can be given. Still, if it were possible for us to give it in the red-hot state, the action would be still more positive and rapid, for in the cooling the charcoal will absorb the gases that are in a normal atmosphere as well as in an abnormal atmosphere. The object in heating it is to drive off all those gases that are in the charcoal, so that it will be as devoid of gaseous matters as it is possible to have it. It makes no difference what the gases are that are in it, for the less gas it has in its structure the more will it absorb, and *vice versa*. However, I do not think it advisable for us to administer it in the red-hot state, especially if the owner be present, and our case terminate fatally, for the capsule might dissolve in the pharynx, and with expirations the animal would exhale sparks of fire, and if the latter takes place, the probabilities are that we would have to defend a suit for damages.

